# Structural analysis of melanosomes in living mammalian cells using scanning electron-assisted dielectric microscopy with deep neural network

**DOI:** 10.1016/j.csbj.2022.12.027

**Published:** 2022-12-18

**Authors:** Tomoko Okada, Tomoaki Iwayama, Taku Ogura, Shinya Murakami, Toshihiko Ogura

**Affiliations:** a Health and Medical Research Institute, National Institute of Advanced Industrial Science and Technology (AIST), Central 6, Higashi, Tsukuba, Ibaraki 305-8566, Japan; b Department of Periodontology, Osaka University Graduate School of Dentistry, 1-8 Yamada-oka, Suita, Osaka 565-0871, Japan; c Chemical Business Unit, Nikko Chemicals Co., Ltd., Itabashi-ku, Tokyo 174-0046, Japan

**Keywords:** Scanning electron-assisted dielectric microscopy, Raman microscopy, Melanosome, Melanocyte, MNT-1 cell, Deep neural network

## Abstract

Melanins are the main pigments found in mammals. Their synthesis and transfer to keratinocytes have been widely investigated for many years. However, analysis has been mainly carried out using fixed rather than live cells. In this study, we have analysed the melanosomes in living mammalian cells using newly developed scanning electron-assisted dielectric microscopy (SE-ADM). The melanosomes in human melanoma MNT-1 cells were observed as clear black particles in SE-ADM. The main structure of melanosomes was toroidal while that of normal melanocytes was ellipsoidal. In tyrosinase knockout MNT-1 cells, not only the black particles in the SE-ADM images but also the Raman shift of melanin peaks completely disappeared suggesting that the black particles were really melanosomes. We developed a deep neural network (DNN) system to automatically detect melanosomes in cells and analysed their diameter and roundness. In terms of melanosome morphology, the diameter of melanosomes in melanoma cells did not change while that in normal melanocytes increased during culture. The established DNN analysis system with SE-ADM can be used for other particles, e.g. exosomes, lysosomes, and other biological particles.

## Highlights

• Direct observation of melanosomes in living cells by SE-ADM.
• Melanosomes of MNT-1 cells have a toroidal structure.
• Shape of melanosomes differs depending on cell type.
• DNN enables highly accurate detection of melanosomes in SE-ADM images.

## Graphical Abstract

**Figure:**
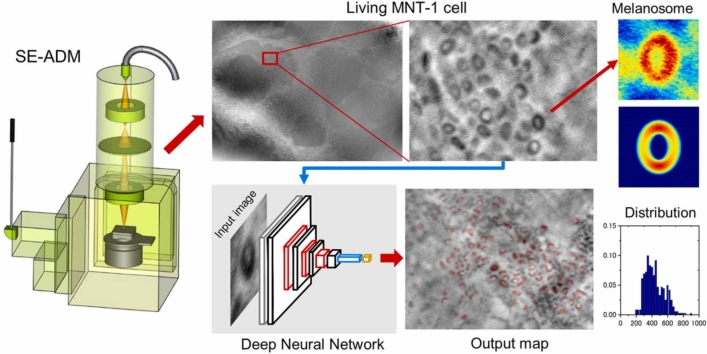

## 1 Introduction

Melanins are the main pigments in mammalian skin and hair. Physical and biological stress promotes the release of stress signals in the skin which may modulate the synthesis of melanins through the release of hormones [1]. Melanins are synthesised in intracellular compartments called melanosomes [2], [3], [4]. It is known that melanosomes develop through four morphologically distinct stages [2], [3], [4], [5]. Stage I melanosomes are conventional vacuolar early endosomal compartments displaying a planer clathrin coat and few intraluminal vesicles. In Stage II melanosomes, fibrils elongate and assemble laterally into fibrillar sheets. In Stage III melanosomes, melanin synthesis begins. Stage IV melanosomes contain enough melanin to mask the underlying fibrillar structure [2], [3], [4], [6], [7].

Melanin can be found in two forms, eumelanins (black and brown pigments) and pheomelanins (red and yellow pigments) [8]. Mammalian melanin is a complex of these two types of melanin, and their ratios are responsible for the differences in skin and hair colour. Both eumelanin and pheomelanin are synthesised via a series of reactions that are initiated by the oxidation of tyrosine to dopaquinone, catalysed by the enzyme tyrosinase (TYR) [3], [8], [9], [10]. TYR is a Cu^2+^-binding protein, which oxidises its substrates tyrosine and L-dopa to generate dopaquinone [6].

TPC2 (two-pore channel 2) is a gated cation channel which is activated by phosphatidylinositol 3,5-bisphosphate. TPC2 is ubiquitously expressed and localized on melanosomes and on endosomes and lysosomes. Knockdown of TPC2 in mouse melanocytes results in increased melanin content and melanosome size suggesting that TPC2 negatively regulates melanosome biogenesis [11].

Recently, we developed a novel imaging technology named scanning electron-assisted dielectric microscopy (SE-ADM) based on scanning electron microscopy (SEM), which enables the observation of various biological specimens in aqueous conditions without radiation-induced damage [12], [13]. A biological sample is enclosed in a hand-made sample holder composed of two silicon nitride (SiN) films. The upper SiN film is coated with tungsten (W). When an electron beam (EB) is applied to the tungsten-coated SiN film, EB is absorbed and causes a local potential change. This potential change propagates well through water, having a high dielectric constant of 80. On the other hand, a substance such as an amino acid that is a constituent of a protein, which has low polarisation and has a low dielectric constant of 2–3, greatly attenuates the electric potential transmission. In SE-ADM, the difference in the dielectric constant of the sample can be observed as the image contrast, where substances with high dielectric constant are observed to be light while substances with low dielectric constant such as amino acids that constitute proteins are observed to be dark [12], [13]. Our SE-ADM system enable to acquire high-contrast nano level imaging of living cultured mammalian cells under aqueous conditions without fixation and staining [14], [15], [16], [17].

In this study, we focused on the observation of melanosomes using our SE-ADM system. Because melanins (eumelanins) are very dark, we expected that our SE-ADM system would be advantageous for their observation. Further, we have successfully analysed the morphology of melanosomes in living cells using automatic recognition by a deep neural network (DNN). This is based on the automatic recognition system for protein particles in electron microscopy that we had previously developed based on a three-layer artificial neural network (ANN) [18], [19]. Here, we used a modern DNN system [20] to recognise melanosomes from SE-ADM images with high accuracy and analysed their structural information. In addition, the spectra of melanosomes in living cells were detected and analysed by Raman microscopy.

## 2 Results

### 2.1 Observation of melanosomes in MNT-1 cells using SE-ADM

**Fig. 1:**
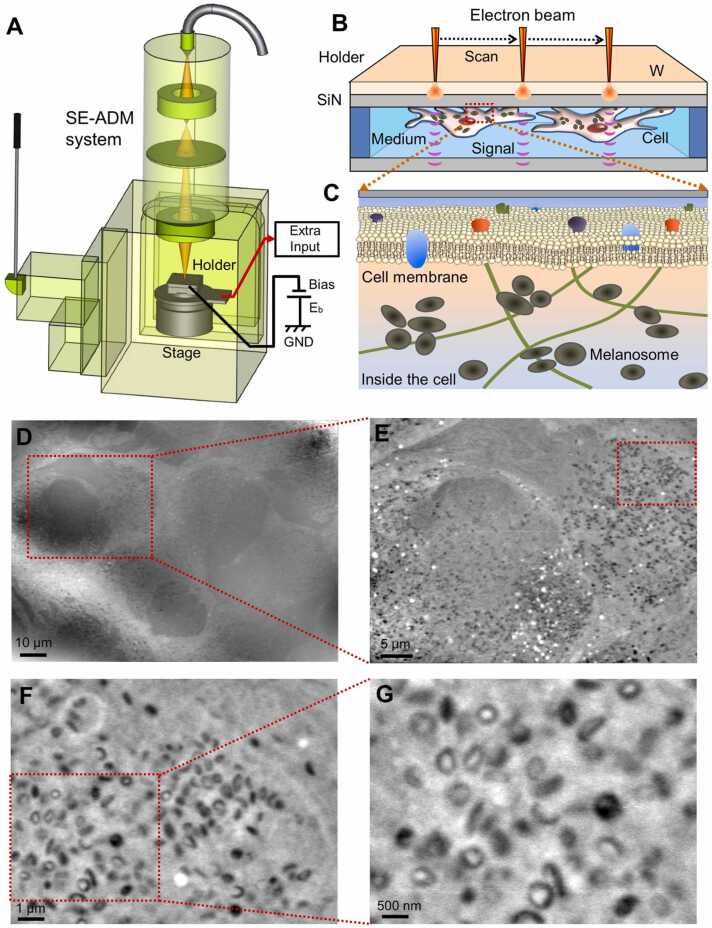
Experimental setup of SE-ADM system and observation of cultured melanoma cells. A) A schematic diagram of the SE-ADM system based on high resolution FE-SEM. The liquid-sample holder containing cultured cells was mounted on the stage attached to the pre-amplifier, which was introduced into the SEM specimen chamber. The scanning EB was applied to the W-coated SiN film at a low acceleration voltage. The measurement terminal under the holder detected the electrical signal through the liquid specimens. B) Overview of the liquid-sample holder with cultured melanoma cells. MNT-1 cells were on the upper SiN film and the W-coated side was irradiated with the scanning EB. C) A conceptual diagram of melanosomes in a cell. D) A low magnification SE-ADM image of living MNT-1 cells 7 days after the culture in medium (1000 ×) with a 6-kV EB and − 9 V bias. E) An SE-ADM image of MNT-1 cells (2500 ×) in the red rectangle in (D). F) A high magnification image of MNT-1 cells (10,000 ×). G) A higher magnification image (20,000 ×) of melanosomes in the red rectangle in (F). Scale bars, 10 µm in (D), 5 µm in (E), 1 µm in (F) and 500 nm in (G).

MNT-1, a human melanoma cell line [9], was cultured on a SiN film of 50 nm thickness in a culture dish holder [14]. After cells formed a subconfluent monolayer on the SiN film, the holder was separated from the plastic dish, sealed in an acrylic holder, and installed in the SE-ADM system (Fig. 1A–C). Cultured MNT-1 cells in the holder were kept under atmospheric pressure. The nucleus was clearly observed in a low magnification image (1000 ×, Fig. 1D). It was observed that high density black particles were dispersed in the cells (Fig. 1E–G). The cell pellets consisting of cultured MNT-1 cells appeared black after centrifugation. Therefore, we consider that the black particles in the images of SE-ADM represent melanosomes in the melanoma cells. The distribution of melanosomes was not homogeneous. All the melanosomes were localized in the cytoplasm but not in the nucleus.

**Fig. 2:**
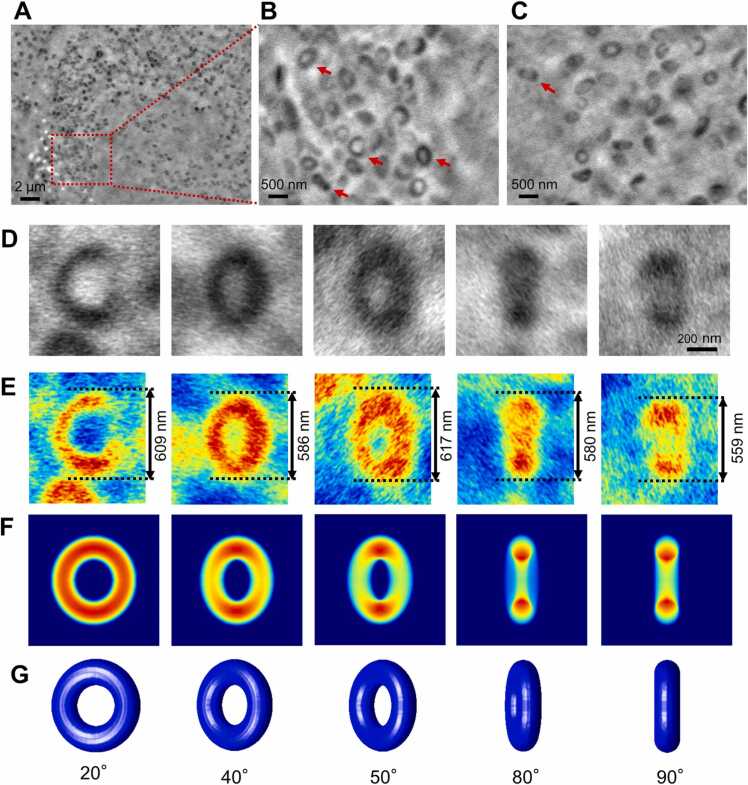
SE-ADM image of MNT-1 after 4 days of culture. A) SE-ADM image of melanosomes in MNT-1 cell (5000 ×) with an 8 kV EB. B) A high magnification image (20,000 ×) of melanosomes in the red rectangle in (A). Many of the melanosomes were clearly observed as a toroidal shape. C) A high magnification image (20,000 ×) of melanosomes in another area. D) Enlarged melanosome images indicated by red arrows in (B) and (C). E) Pseud-coloured melanosomes of (D). The mean diameter of the melanosomes was 590 ± 23 nm. F) Pseudo-colour projection images of the 3D model predicted from melanosome images of (E). G) The prediction 3D model images of melanosomes. The angle of each melanosome with respect to the axis of the EB was indicated. Scale bars, 2 µm in (A), 500 nm in (B) and (C), and 200 nm in (D).

We then analysed the melanosome images in SE-ADM in more detail. The images of melanosome particles selected in MNT-1 cells were enlarged (Fig. 2A–D). The average diameter of melanosome particles estimated from five particles in the elongated images of Fig. 2D and E was about 590 ± 23 nm, which is consistent with the size reported previously [4], [21]. Interestingly, most of these formed annulus structures with low dielectric constant at the centre and elongated. From these images, the 3D structure of melanosomes is predicted to be toroidal (Fig. 2F, G). The toroidal structure varies from a doughnut shape to an elongated structure depending on the angle of view. The viewing angles of the predicted toroidal structures and their projected images for each of these melanosome images are shown in Fig. 2F and G. Previous studies reported that the structure of melanosomes had an elongated spherical structure [22]. However, the shape of melanosomes in MNT-1 cells observed by SE-ADM differs from that of previous reports. In our SE-ADM system, since we did not fix or stain the cells but observed the live cells in the medium, the toroidal morphology of melanosomes seems to be a natural form.

### 2.2 Detection of melanosomes inside MNT-1 cells and genetically modified MNT-1 cells using confocal Raman microscopy

**Fig. 3:**
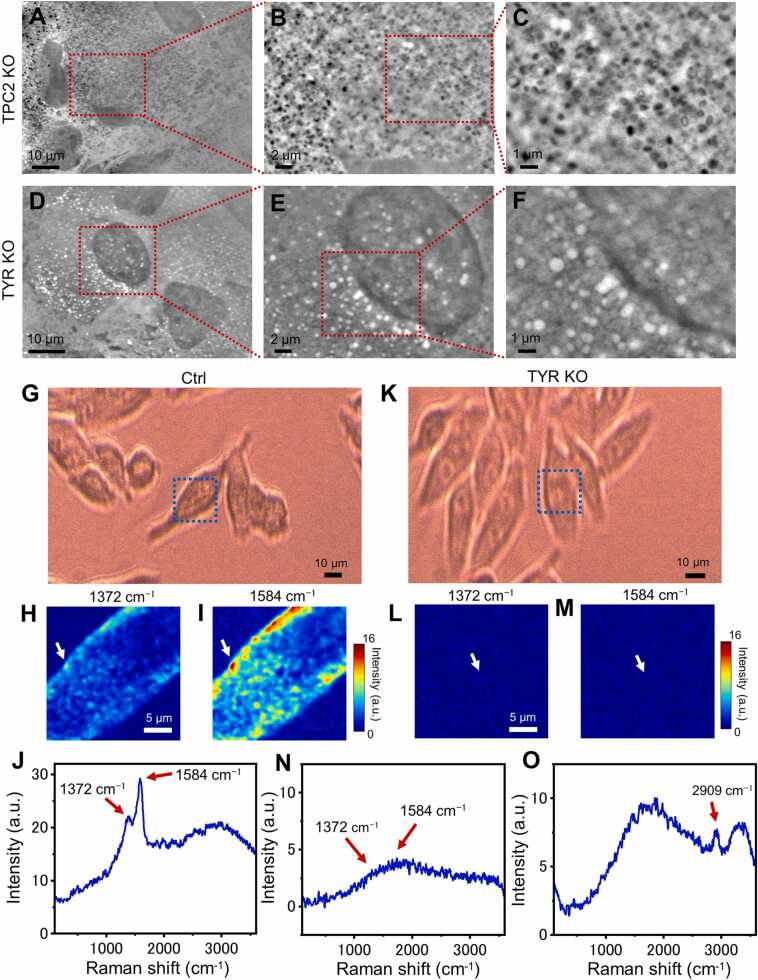
Spectrum analysis of MNT-1, TPC2 and TYR knocked down MNT-1 using confocal Raman spectrum microscope. A−C) SE-ADM images of TPC2 knocked down MNT-1 cells at 1500 × , 5000 × and 10,000 × magnifications with an 7 kV EB. Increased number of melanosomes compared to parent MNT-1 cells were observed. D−F) SE-ADM images of TYR knocked down MNT-1 cells at 2000 × , 5000 × and 10,000 × magnifications. In the TYR knocked down MNT-1 cells black particles completely disappeared. G) An optical microscopic image of living MNT-1 cells. H) A 2D Raman image of the blue square in (G) at the carbon peak of 1372 cm^-1^ by 0.2 mW laser intensity and 50 ms measurement time at each pixel. I) A 2D Raman image at the carbon peak of 1584 cm^-1^. J) Raman spectra of the high carbon peak of MNT-1 indicated by a white arrow of (H) and (I). Clear peaks at 1372 and 1584 cm^-1^ were detected which corresponded to the reported melanin peak. K) An optical microscopic image of TYR knocked down living MNT-1 cells. L) A 2D Raman shift image of the blue square in (K) at the carbon peak of 1372 cm^-1^. The laser intensity was 0.2 mW. M) A 2D Raman shift image at the carbon peak of 1584 cm^-1^. No structures can be seen in the carbon peak images. N) Raman spectra at the centre of cell indicated by a white arrow of (L) and (M). O) Raman spectra at the centre of another cell at 2 mW laser intensity. In this plot, the lipid peak of 2909 cm^-1^ is clearly visible. Scale bars, 10 µm in (A, D, G, K), 2 µm in (B, E), 1 µm in (C, F) and 5 µm in (H, L).

To confirm whether the black particles of MNT-1 by SE-ADM were indeed melanosomes, we observed cells in which genes related to melanosome production were suppressed. We knocked-down the expression of TPC2 and TYR gene in MNT-1 cells using a CRISPR-CAS9 system and established two TPC2 KO clones and two TYR KO clones. In the TPC2 KO clone cells, more black particles were observed in the cells (Fig. 3A–C) compared to the parent MNT-1 cells, while no black particles were observed in the TYR KO cells (Fig. 3D–F). The two TYR KO clones and two TPC2 KO clones showed the same result. This result demonstrated that the black particles in the SE-ADM images were melanosomes.

We then examined whether the black particles observed by SE-ADM in MNT-1 cells were really melanosomes or not using MNT-1 and TYR KO cells with a confocal Raman microscope. As shown in Fig. 3G−J, the Raman spectrum of melanosome in MNT-1 cells showed a sharp peak at 1372 and 1584 cm^-1^ using 0.2 mW laser intensity which corresponded to the melanin peaks reported previously [23], [24], [25]. Furthermore, the Raman peak images of MNT-1 showed the distribution of melanosomes inside the cells (Fig. 3H, I). When we measured the cells at laser intensities higher than 0.2 mW, the melanosomes were broken and a broad fluorescence spectrum was detected in the cells. We therefore reduced the laser intensity to 0.2 mW. On the other hand, the carbon peak was completely diminished in the TYR KO cells at 0.2 mW laser intensity (Fig. 3K−N). In TYR KO cells, there were no melanosomes, so we increased the laser intensity to 2 mW. When Raman measurements were performed with a laser intensity of 2 mW, a lipid peak of 2909 cm^-1^ [26], [27] was observed without carbon peak (Fig. 3O). Therefore, we proved that the very dark particles in MNT-1 cells correspond to melanosomes containing melanin.

We then examined if there was lipid at the centre of the toroidal shape of the melanosome. In our SE-ADM system, high density and low-dielectric samples show clear contrast [28], [29]. Protein particles are black and lipids are white, as shown in our previous report [28], [29]. Therefore, there was a possibility that there were some lipids at the centre of the toroidal shape of the melanosomes. To investigate this, we averaged the strong Raman signal points of 1372 cm^-1^ and 1584 cm^-1^ of melanosome and checked for lipid Raman peaks (Supplementary Fig. 1). Here, Raman images of 1372 cm^-1^ and 1584 cm^-1^ were added (Supplementary Fig. 1A), and pixels with an intensity of 10 or more were extracted from the added image (Supplementary Fig. 1B). Furthermore, Raman spectra of these selected pixels were averaged (Supplementary Fig. 1C), the noise was greatly reduced and the carbon peaks were seen clearly (Supplementary Fig. 1D). However, under this condition, the lipid peak at 2909 cm^-1^ was not observed even in the enlarged plot (Supplementary Fig. 1E). Therefore, it is expected that lipid is absent in the central part of the melanosome in the toroidal structure.

### 2.3 Observation of melanosomes in normal human melanocytes and other melanoma cells using SE-ADM

**Fig. 4:**
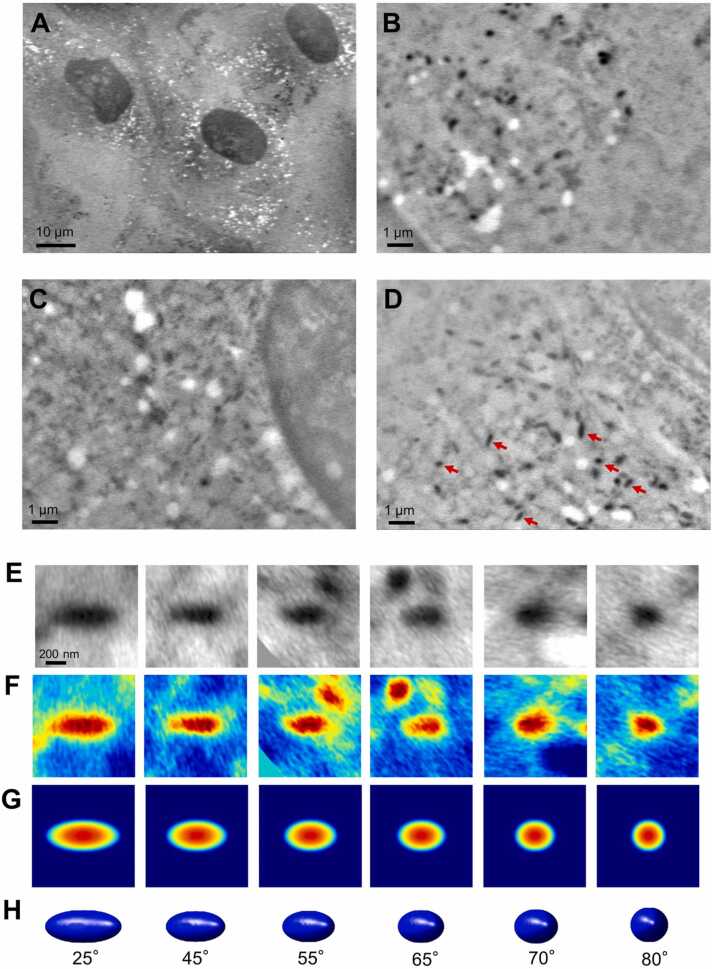
SE-ADM image of normal melanocytes after 5 days of culture. A) A low magnification (1500 ×) SE-AMD image of melanosomes in normal melanocytes with a 7 kV EB. B−D) A high magnification image (10,000 ×) of melanosomes in normal melanocytes. E) Enlarged melanosome images in the normal melanocytes indicated by red arrows in (D). F) Pseudo-coloured melanosomes of (E). G) Pseudo-coloured projection images of the 3D model predicted from melanosome images from (F). H) The prediction 3D model images of melanosomes of (F). The angle of each melanosome is indicated. Scale bars, 10 µm in (A), 1 µm in (B−D) and 200 nm in (E).

Normal human neonatal melanocytes were cultured on a SiN film of 50 nm thickness in a culture dish holder as mentioned above. Interestingly, the shape of the melanosome was not toroidal, but ellipsoidal (Fig. 4A−D), as reported previously [2], [4], [22]. We selected some melanosomes and analysed them in detail (Fig. 4E − H). The melanosomes are distributed in the cytoplasm.

MNT-1 cells and normal melanocytes were cultured in different media. Since the cell morphology is affected by the culture medium, we have cultured MNT-1 cells in the medium used for the melanocytes and observed them by SE-ADM. In this case, the shape of melanosomes was the same as that when cultured in the medium for MNT-1 (Supplementary Fig. 2). Further, we have observed human adult melanocytes in addition to human neonatal melanocytes and obtained almost the same results. The average of the major axis of melanosome was 482 ± 67 nm (Supplementary Fig. 3). We have also observed B16 melanoma cells by SE-ADM and found that the melanosomes in these cells were not toroidal but ellipsoidal (Supplementary Fig. 4). We presume that the shape differs according to cell type.

### 2.4 Detection of melanosomes using a DNN system

We have developed a DNN system to automatically detect melanosomes in cells and we measured and analysed their length and circularity. We previously developed an automatic detection system for protein particles in electron microscopy images using a three-layer pyramidal type ANN [18], [19]. Here, a multi-layer ANN system including a convolutional layer was applied for more accurate particle recognition and mask processing [20], [30], [31].

**Fig. 5:**
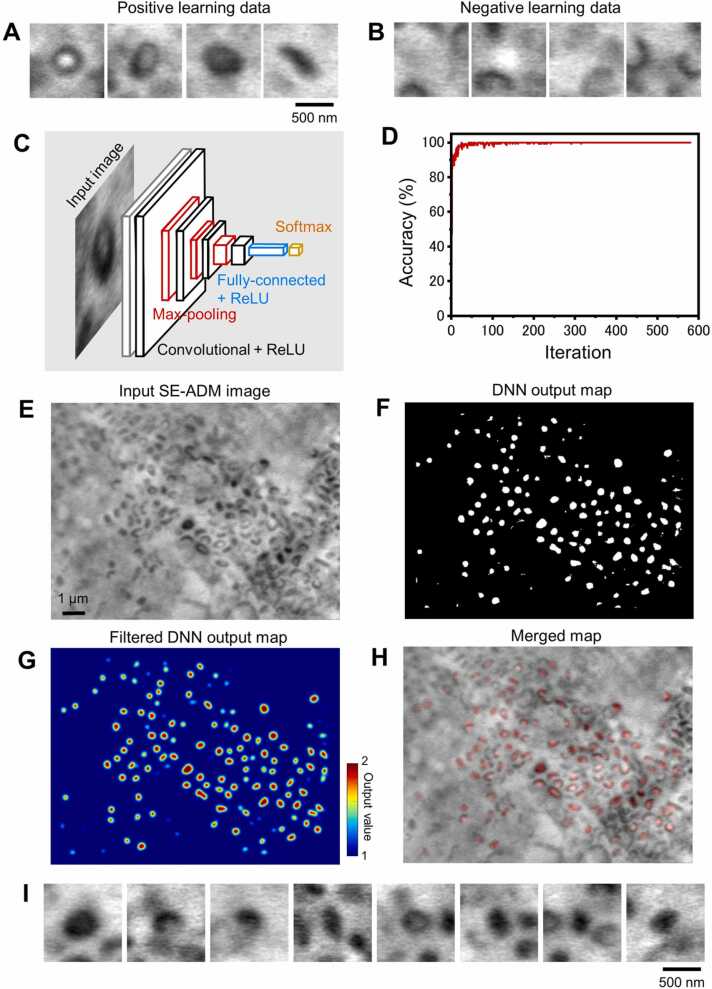
Detection and analysis of melanosomes using the DNN system. A) Typical melanosome images of MNT-1 observed by SE-ADM. We manually selected 239 typical images. These images were given as positive learning data to the DNN system. B) Typical non-melanosome images observed by SE-ADM. We manually selected 307 non-melanosome images in the cell. These images were given as negative learning data to the DNN system. C) A schematic diagram of the DNN system. The DNN consists of an input layer, the convolutional layers, Max-pooling layers, and a fully-connected layer with ReLU, and the final output layer is the softmax layer. Details of the DNN are described in Materials and Methods. D) Recognition accuracy for each learning iteration calculated from unlearned 1000 images. After 50 trials, the accuracy reached quite close to 100 %. E) Typical SE-ADM image of MNT-1 cells (10,000 ×) inputs to the DNN system. F) DNN output map for the input image in (E). The output value at the particle position is 2 and 1 otherwise. G) Gaussian filtered DNN output map. The kernel size of the filter is 25 × 25 pixels with σ = 7. H) Merged image of the input image (E) and the Gaussian filtered DNN output (G). Red areas indicate locations of high DNN output. I) Typical SE-ADM melanosome images in MNT-1 cells automatically picked up using the filtered DNN output map of (G). Scale bars, 500 nm in (A, I) and 1 µm in (E).

This DNN is composed of an input layer, a convolutional layer, and a Max-pooling layer [20], [30], [31]. These are stacked in four layers, and this output serves as the input to the fully-connected layer and the softmax layer to obtain the final output (Fig. 5C). The SE-ADM image was input into the input layer resulting in an output of 2 for melanosome images and 1 for other background and intracellular structures. We first selected 239 melanosome images as a positive learning dataset (Fig. 5A), and selected the 307 background images in which there were no melanosomes at the centre, as a negative learning dataset (Fig. 5B). All training data including positive and negative images were rotated in-plane in 10-degree increments to increase the number of training data 36-fold to 19,656. From this, 1000 images were randomly selected as data for accuracy verification, and the remaining 18,656 images were used as training data. We have previously succeeded in recognising protein particle images with high accuracy by training similar particle images and procedure on a pyramidal type ANN [18], [19]. We then established a new detection system using the DNN (Fig. 5C). The DNN learned 18,656 training images in random order, and this was repeated 580 times. At the end of each learning cycle, accuracy calculations were performed using 1000 untrained images for accuracy verification (Fig. 5D). Over 50 iterations of this training, the calculated accuracy is fairly close to 100 %.

In the calculation of the DNN output map of the SE-ADM image, the SE-ADM image of the MNT-1 cell (Fig. 5E) is cropped at the pixel size of the input layer, shifting the position in order from the upper left edge, and this is input to the learned DNN. The output value of this DNN is output to the corresponding position in the output map (Fig. 5F). The output map of the original DNN consists of binary values, 1 for the background (black area) and 2 for the particle positions (white area) (Fig. 5F). A broad Gaussian filter is applied to this DNN output map to obtain the centre of the particle position (Fig. 5G). In the merged image of the DNN output map and the input SE-ADM image, it can be seen that the DNN output is higher at most particle positions (Fig. 5H). By detecting the peak position of the Fig. 5H image, the particle image of melanosomes in the cell can be automatically cut out with high accuracy (Fig. 5I).

### 2.5 Automatic masking process of MNT-1 melanosome images by a DNN

**Fig. 6:**
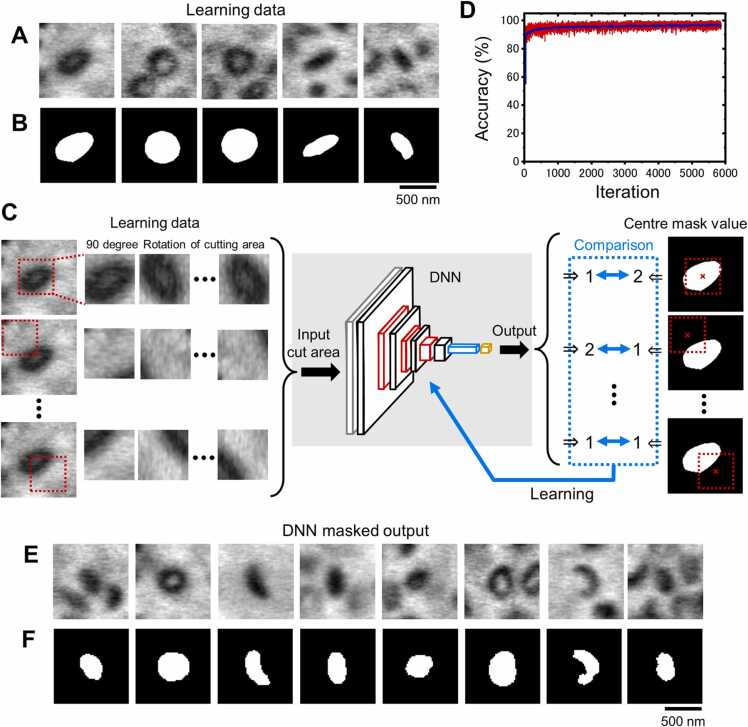
Automatic masking system of melanosome images by DNN. A) Typical SE-ADM melanosome images in MNT-1 cells (10,000 ×). We manually selected 190 typical images for masking. B) Hand-written masking results from SE-ADM images in (A). C) Schematic diagram of the automatic masking system by DNN. The DNN configuration is the same as in Fig. 5C, with an input range of 50 × 50 pixel. Details of the masking DNN are described in Materials and Methods. D) Recognition accuracy for each learning iteration calculated from unlearned 2000 images. The red line indicates the training accuracy of each cycle and the blue line indicates the smoothed training accuracy. After 1000 trials, the accuracy reached close to 97 %. E) Typical SE-ADM melanosome images in MNT-1 cells (10,000 ×) picked up by the trained DNN system. F) Automatic masking results of melanosomes using the trained DNN system. Scale bars, 500 nm in (B, F).

We then masked and binarised the melanosome areas to calculate the length and circularity of the detected particles. To automatically mask the images, using approximately 190 melanosomes, we manually masked the images of melanosomes (Fig. 6A, B) in order to train the DNN system using these images. Each masked training dataset was rotated in-plane every 90° and increased to 1000 images by randomly shifting the position by± 25 pixels. This brings the total training data to 190,000 images, from which 2000 images were randomly extracted for accuracy verification. The DNN was trained with the melanosome training images as input and with the central mask value of cut area as output (Fig. 6C). The accuracy of the masking process was close to 97 % after learning iteration of 1000 times (Fig. 6D). The system performed the masking process and drew the masked images. Input of melanosome images to the mask-trained DNN produced highly accurate mask images (Fig. 6E, F).

The U-net structure is often used in the identification of regions of an image, which can identify regions of the entire input image with high accuracy [32]. However, this process requires masking only the particles in the centre of the cropped melanosome image and excluding those at the edges. In order to perform such mask processing with high accuracy, the processing by DNN proposed here is suitable.

### 2.6 Comparison of melanosome morphology in the MNT-1 cells and normal melanocytes

**Fig. 7:**
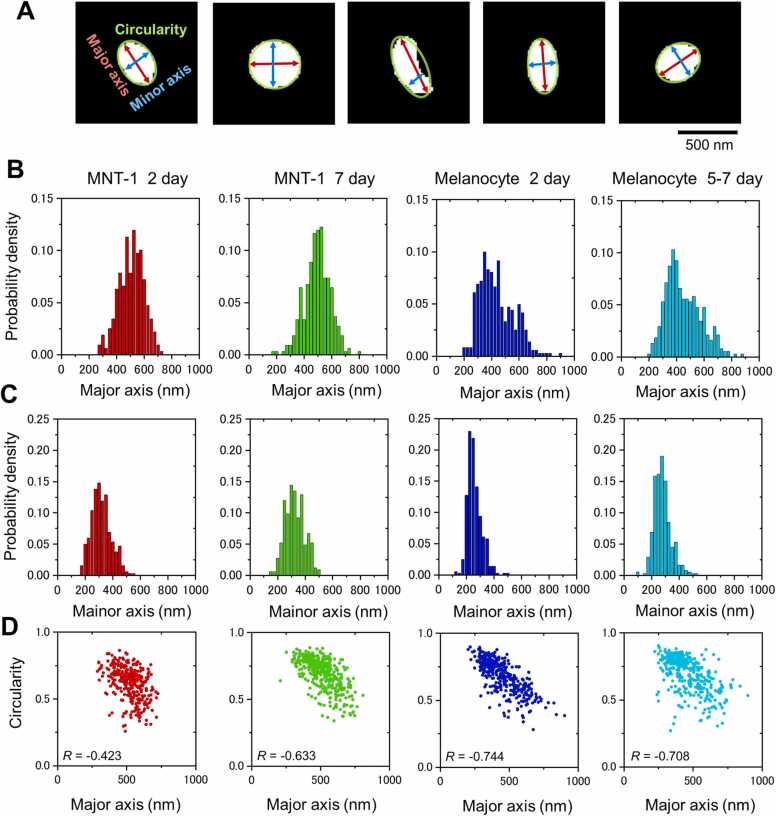
Comparison analysis of the morphology between melanosomes in MNT-1 cells and normal melanocytes. A) Schematic of the calculation of the major and minor axes and circularity of the melanosomes using the DNN masked output images. The red and blue arrows indicate the major and minor axes, and the green circle indicates the circularity. B) Comparison of major axis. The major axis of melanosomes in MNT-1 had a peak at around 500 nm and did not change between day 2 and day 7 of culture. On the other hand, the major axis of melanosomes in normal melanocytes showed a big peak at 300–400 nm and a small peak at 500–600 nm and on day 7, a peak emerged at 700 nm. The average and standard deviation of the major axis of each melanosome are 519 ± 90 nm on day 2 of MNT1, 512 ± 108 nm on day 7, 448 ± 125 nm on day 2 of melanocyte, and 462 ± 125 nm on day 5–7. C) Comparison of minor axis. The minor axis of melanosomes in MNT-1 showed a peak at 300 nm and this did not change on day 2 and day 7 of culture. On the other hand, the minor axis of melanosomes in melanocytes showed a peak at 250 nm on day 2 and the peak shifted close to 300 nm on days 5–7. The average and standard deviation of the minor axis of each melanosome are 329 ± 72 nm on day 2 of MNT-1, 318 ± 66 nm on day 7, 272 ± 66 nm on day 2 of melanocyte, and 291 ± 62 nm on day 5–7. D) Comparison of circularity. Neither melanosomes in MNT-1 nor those in normal melanocytes changed their circularity during the culture. The correlation coefficient of each sample is indicated in the graph.

We precisely analyzed the morphology of melanosomes in MNT-1 cells and normal melanocytes using masked images from the DNN detection system described above. Using the masked image, we calculated the major and minor axes and circularity of the particles by binarisation analysis (Fig. 7A). As for the major axis of MNT-1 melanosomes, the peak was not changed between day 2 and day 7 of culture. There was a peak at around 500 nm in both cases. On the other hand, there were two peaks of 300–400 nm and 500–600 nm in normal melanocytes, and a peak at 700 nm emerged on day 7 of culture (Fig. 7B). As for the minor axis, there was a peak at around 300 nm in MNT-1 on both day 2 and day 7 (Fig. 7C), while there was a peak at around 250 nm on day 2 and the peak was shifted to 300 nm on day 7 in the normal melanosomes. This means that the diameter of melanosomes in MNT-1 did not change but that of the melanosomes in normal melanocytes rather increased during culture. The circularity of MNT-1 on the second day of culture is widely distributed in the range of 0.8–0.25 when the major axis is 500 nm or less (Fig. 7D). This suggests that even small melanosomes contain a mixture of elongated and round particles. At day 2 of melanocyte culture, longer melanosomes have lower circularity. However, after day 7 of incubation, the distribution of circularity in MNT-1 and melanocytes is similar. The correlation coefficients between circularity and major axis are *R* = −0.423 on day 2, − 0.633 on day 7 for MNT-1, and − 0.744 on day 2 and − 0.708 on day 5–7 for melanocyte.

### 2.7 Toroidal morphology of melanosomes secreted from MNT-1 cells

To examine the morphology of melanosomes secreted by MNT-1, we observed the supernatant of MNT-1 cells by SE-ADM. The shape of secreted melanosomes was not ellipsoidal but toroidal (Supplementary Fig. 6). Therefore, the toroidal structure of MNT-1 cells is retained even after secretion in the medium.

## 3 Discussion

In our SE-ADM system, samples in a culture dish holder are enclosed by two SiN films and the cells inside the holder can be kept under atmospheric pressure [14], [15]. Further, because the EB is absorbed by the W layer which coats one side of the SiN film, cells can escape damage by the EB. As a result, we can observe cells without fixation and staining [14], [15].

In this study, we observed melanosomes in living human melanoma cells MNT-1 and normal melanocytes. The melanosomes were clearly discerned as black particles (Fig. 1, Fig. 2). We proved that they were melanosomes using confocal Raman microscope (Fig. 3). The peaks of 1372 and 1584 cm^-1^ correspond to the peaks of melanin previously reported (Fig. 3J). Moreover, when we knocked down the expression of tyrosinase, both the black particles in the images and the peaks of melanin were completely diminished (Fig. 3D−F, K−N). Interestingly, the morphology of many melanosomes in melanoma MNT-1 is toroidal (Fig. 1, Fig. 2). However, melanosomes of normal human melanocytes (neonatal and adult) are ellipsoidal rather than toroidal (Fig. 4, Supplementary Fig. 3). The reason for the difference in morphology is not clear at present. In previous reports using isolated melanosomes and/or fixed cell sections, images of toroid-shaped melanosomes have been detected in human melanoma MNT-1 [9], [21], in zebra fish [33] and in mouse melanoma [34]. Therefore, the toroidal shape might be of significance in the process of melanosome biogenesis. However, in previous studies they did not mention about the toroidal structure of the melanosomes.

There are some differences between the SE-ADM particle images and the transmission images of the 3D toroidal structure. One reason for this discrepancy is that the model structure has a toroidal structure in empty space, whereas the SE-ADM image has various intracellular organelles and proteins near the melanosomes. Furthermore, it is expected that the toroidal structure of the melanosomes inside the cells is slightly distorted or bent. For this reason, it is thought that when the particles are viewed as an elongated image at an angle of 90°, there is a shift between the front side and the back side, the area of the upper and lower bright spots changes, and the contrast intensity also decrease.

In our SE-ADM, an EB is applied to a W-coated SiN film, and this potential signal is detected by a metal terminal under the sample holder after passing through the sample. Therefore, the SE-ADM image is a transmission image of the cells. Spatial resolution is high in the area close to the SiN film on the upper side, but the resolution decreases slightly toward the lower side. The MNT-1 and melanocytes used in this study adhere well to the SiN film and are thin, so the spatial resolution does not decrease so much. Therefore, the effect of depth on the resolution of melanosome images is expected to be small.

Observation of normal cells by SE-ADM shows cell nuclei with extremely dark contrast. This diameter is around 10–20 µm [14]. The cell nucleus contains more DNA, RNA, and various proteins than the cytoplasm, resulting in a dark contrast compared to the cytoplasm. On the other hand, in the observation of MNT-1 and melanocytes, the contrast of melanosomes is equal to or higher than that of cell nuclei. As a result, the relative contrast of the nuclei is reduced, compared to normal cells. In TYR KO cells, which do not produce melanin pigment, the cell nuclei are observed with high contrast, almost the same as that in the normal cells (Fig. 3D−F). This result suggests that melanosomes are extremely densely packed with proteins and pigment molecules.

Recently, AFM-based scanning dielectric force volume microscopy has been developed [35]. This method is useful for observing the difference in the dielectric constant under the cantilever at the nano level. However, the observed cells are dehydrated with alcohol after the fixation treatment and are in a dry state.　In our study, the living cultured cells in medium were directly observed by SE-ADM. Therefore, the structure of dried and fixed cells may differ from that of living cells. Recently, we are developing a new impedance microscope that is an improved dielectric microscope [36], [37]. With this method, the impedance information of the sample can be observed by scanning EB while applying an alternating potential signal from below the sample and detecting the impedance signal. In the future, we plan to obtain cell impedance information using our newly developed method*.*

We have developed an analysis method using a DNN particle detection system and auto masking system (Fig. 5, Fig. 6). Using this system, we examined the morphology of melanosomes in human melanoma and melanocytes by automatic masking images from SE-ADM images (Fig. 7). We found that the morphology of melanocytes was changed and that the diameter of melanosomes in melanocytes increased during culture, while the morphology of melanosomes in melanoma did not (Fig. 7). Thus, we have successfully observed melanosomes using our SE-ADM and analysed their morphology with a DNN particle detection system.

DNNs for general image classification consist of more than 20 convolution layers [38], and recently network structures with more than 100 layers have been used　[39]. The reason why the multilayering needs more than 20 convolution layers is that it is for classification of complex images such as animal species and object types. On the other hand, the DNN used in this study has four convolution layers, which is a much smaller network than the conventional DNN used for general image classification. Because the structure of melanosomes is relatively simple, a high recognition accuracy can be achieved with only 4 convolution layers. The same recognition accuracy was achieved with three convolution layers but the recognition accuracy slightly decreased to 98% when two convolution layers were used.

The DNN particle detection system and auto masking system which we have developed here can be used to analyse other particles, for example, extra cellular vesicles, lysosomes and many other inorganic particles.

## 4 Conclusion

We have directly observed melanosomes in living mammalian cultured cells in a medium using SE-ADM. We successfully observed melanosomes as clear black particles and proved that they contained melanin using Raman microscopic analysis. We also developed a DNN system to automatically detect melanosomes in cells and analysed their major and minor axis, and circularity. Using this DNN system, we found that the major axis of melanosomes in normal melanocytes increased during culture.

## 5 Materials and methods

### 5.1 Cell culture

MNT-1 a human melanoma cell line [9], was obtained from ATCC (CRL-3450). MNT-1 cells were cultured in D-MEM (Thermo Fisher Scientific #11995065) containing 10 % AIM-V (Thermo Fisher Scientific #31035025), 10 % MEM NEAA (Thermo Fisher Scientific #10370021), and 20 % fetal bovine serum (Thermo Fisher Scientific) under normal cell culture conditions (37 °C, 5 % CO_2_). Human epidermal melanocytes (isolated from moderately pigmented neonatal foreskin, newborn, 14 days or less) were purchased from Thermo Fisher Scientific (# C1025C) and cultured in Medium 254 (Thermo Fisher #M254500) containing HMGS growth supplements (Kurabo, Japan, #KM-6350, containing FBS, insulin, hydrocortisone, human FGFβ, heparin, transferrin, PMA, bovine pituitary extract). Human adult epidermal melanocytes, NHEM(AD) (KM-4109) were purchased from Kurabo Industries Ltd. (Osaka, Japan) and cultured in DermaLife Basal Medium (LKB-LM0004) containing DermaLife M LifeFactors kit (LMK-LS1041) including insulin, transferrin, epinephrin, etc. B16 mouse melanoma (RCB1283) cells were purchased from Riken BRC cell bank (Tsukuba, Japan) and cultured in DMEM (Fujifilm Wako pure chemical Ltd. Osaka, Japan) containing 10 % FBS (Thermo Fisher Scientific). For SE-ADM imaging, cells (4 × 10^4^, 1.5 mL/dish) were cultured on a 50 nm thick SiN film in a hand-made culture dish holder [14]. After 2–7 days of culture, the cells formed a sub-confluent monolayer on the SiN film in the holder. The cells were cultured under 5 % CO_2_ at 37 °C for 2 days or 5–7 days just before the observation by SE-ADM.

### 5.2 Generation of CRISPR/Cas9-mediated TYR or TPC2 KO MNT-1 clones

TYR or TPC2 KO MNT-1 clones with different target sequences were generated using CRISPR/Cas9 and single-cell sorting as previously described [40]. Both clones yielded similar results. Target-specific crRNA, ATTO550-conjugated trans-activating crRNA, and Cas9 protein were purchased from Integrated DNA Technologies Inc. (IL, USA). The target sequences were as follows: TYR (NM_000372), ATTTGGCCATAGGTCCCTAT (536–555, Exon 1) or GATTGTCTGTAGCCGATTGG (898–917, Exon 2); TPC2 (NM_139075), GCTCGATGTGGCTTTACCGA (272–291, Exon 3) or ATACGTTCGAGTAATACCGT (291–310, Exon 3). Ribonucleoprotein (RNP) complex was formed according to the manufacturer’s protocol, and RNPs were transfected to 1 × 10^5^ MNT-1 cells using the Neon electroporation system (Thermo Fisher Scientific, MA, USA) with 10 μL tips at 1300 V for 10 ms and 3 pulses. Localisation of ATTO550 particles was confirmed on the next day of transfection under a fluorescent microscope (Nikon, Japan). Three days after transfection, single-cell sorting was performed using the SH800Z Cell Sorter (Sony Biotechnology, CA, USA) with the 130-μm microfluidic sorting chips set on a single-cell mode. Cells were sorted into 96-well plates containing the cell culture medium. No multiple colonies per well were found, and a single cell-derived colony was passaged in 24-well plates, 6-well plates, and finally 10 cm dishes. The genomic DNA was obtained during the expansion, and the positive clones were identified by MultiNA (Shimadzu, Kyoto, Japan) capillary electrophoresis of polymerase chain reaction (PCR) amplicons for the targeted genome region.

### 5.3 Liquid sample culture dish holders

The liquid sample holder of the SE-ADM was made as previously described [13], [14]. Briefly, the liquid sample holder comprising an upper Al holder and lower acrylic resin portion held the cell culture solution at atmospheric pressure between the SiN films.

MNT-1, TYR KO MNT-1, TPC2 KO MNT-1 cells, human melanocyte (neonatal and adult) or mouse and human melanoma cells (4 × 10^4^, 1.5 mL/dish) were cultured in the dish holder. These cells formed a sub-confluent monolayer on the SiN membrane in the holder after 2–7 days. Next, the Al holder with a cell monolayer was separated from the plastic culture dish, attached upside down to another SiN film on an acrylic plate and sealed. The Al holder received a voltage bias of approximately − 9 V in the SE-ADM system.

### 5.4 High-resolution SE-ADM system and SEM setup

The handmade SE-ADM imaging system was attached to a field-emission SEM (SU5000, Hitachi High-Tech Corp, Japan) (Fig. 1A). The liquid sample holder was mounted onto the SEM stage and the detector terminal was connected to a pre-amplifier under the holder [13], [14]. The electrical signal from the pre-amplifier was fed into the external input of SEM. The SEM images (1280 × 1020 pixels) were captured at 1000–20,000 × magnification with a scanning time of 40 s, a working distance of 7 mm, an EB acceleration voltage of 6–8 kV and a current of 1–10 pA. High-resolution SE-ADM images were processed from the LPF signal and scanning signal using the image-processing toolbox of MATLAB R2021a (Math Works Inc. Natick, MA, USA). The original SE-ADM images were filtered using a 2D Gaussian filter (GF) with a kernel size of 11 × 11 pixels and a radius of 1.2 s. Background subtraction was achieved by subtracting SE-ADM images from the filtered images using a broad GF (400 × 400 pixels, 200σ).

### 5.5 Raman microscopy

MNT-1 cells were cultured on a glass bottomed dish (Matsunami glass Ltd, Osaka, Japan). The dish was filled with culture medium and sealed with a handmade glass lid and set upside down in the field of the Raman microscope. The cells were observed under a confocal Raman microscope using a 532 nm Nd:YAG laser (alpha300R, WITech, Ulm, Germany). Spectra were acquired with a Peltier-cooled charge-coupled device detector (DV401-BV, Andor, UK) with 600 gratings/mm (UHTS 600, WITec, Germany). The Raman data were analysed by WITec suite (version 5.0, Lab Co., Northampton, MA, USA) and MATLAB R2021a. For observation of MNT-1 cells, a 24 × 24 µm area at the cell centre was scanned at 120 × 120 pixel using a 50 × objective lens at laser intensities of 0.2 mW (Fig. 3h−n) and 2 mW (Fig. 3o) at 50 ms for each pixel.

### 5.6 Automatic detection of melanosomes by DNN

We located melanosomes in dielectric images and analysed their diameter and shape. To detect the position of melanosomes in the observed images, we constructed a DNN with four layers of convolutional layers, batch normalisation layers, rectified linear units (ReLU) and three Max-pooling layers [20], [30], [31]. (Fig. 5C). A fully-connected layer was further added after these layers and finally a softmax layer was used to form the final output [20], [30], [31]. First, 239 melanosome particles were manually selected from the MNT-1 melanosome images as training data, which include everything, i.e. toroidal structures, elongated structures and small particle structures. In the input layer of the DNN, these particle images of 100 × 100 pixels were reduced to a size of 50 × 50 pixels and the intensity values were normalised before input. These images were input into the first layer, and the first and second convolutional layers had 21 and 16 filters with 7 × 7 and 5 × 5 pixel sizes. The Max-pooling layer was set to reduce all three layers to half of their size. The third and fourth convolutional layers have filters of 16 and 32 with 3 × 3 pixel sizes, respectively. The Max-pooling output of the third layer was input to the fully-connected layer of three layers and was input to the two final output units. The final layer, the softmax layer, learns to output a value of 2 for particle image input and 1 for other structures and backgrounds.

The 239 manually selected melanosome images and 307 background images were rotated in-plane in 10-degree increments to increase the number of training data 36-fold to 19,656. From this, 1000 images were randomly selected as data for accuracy verification, and the remaining 18,656 images were used as training data. After reducing the learning images to half pixel size, the DNN was trained to output a value of 2 for the melanosome images and 1 for the other images. The DNN learned 18,656 training images in random order, and this was repeated 580 times. At the end of each learning cycle, accuracy calculations were performed using 1000 unlearned images for accuracy verification. Over 50 iterations of this training, the calculated accuracy is close to 100 % (Fig. 5D).

Observation images of MNT-1 and melanocytes were reduced to 1/2 size and then input into the trained DNN. Since the input size of DNN was 50 × 50 pixel, 50 × 50 pixel was cut out from the upper left edge of the reduced observation SE-ADM image (Fig. 5E), and the output value was sequentially mapped by raster scan (Fig. 5F). A 2D Gaussian filter with a size of 25 × 25 pixel and 7σ was applied to the DNN output map (Fig. 5G, H), and the parts with an output value of 80 % or more were detected (Fig. 5I). The DNN output and data analysis were calculated using MATLAB (R2021a) with the Image Processing Toolbox and Deep Learning Toolbox. DNN training took approximately 2 min on a personal computer (Intel Core i7–9800X, 3.8 GHz and 64 GB RAM, Windows 10).

### 5.7 Automatic mask processing of melanosome images by DNN

Melanosome images detected by DNN can be masked by four layers including convolutional layers, a ReLU and Max-pooling layers. The input layer had a size of 50 × 50 pixels. The four convolutional layers consisted of 35, 21, 16, and 32 filters and 11 × 11, 7 × 7, 3 × 3, and 3 × 3 filter sizes, respectively (Fig. 6C). The output of the fourth layer was input to the fully-connected layer and the final layer, the softmax layer, was trained to output a value of 2 for a particle (melanosome) or 1 for background.

The training data consisted of 190 melanosome images of MNT-1 (200 × 200 pixels) that were manually masked (Fig. 6A, B). This particle training image was cropped at 100 × 100 pixels with random shifting in the range of± 25 and then reduced to 50 × 50 pixels. Each cutting particle image was rotated in-plane every 90° and the total increased to 1000 images (Fig. 6C). The DNN was trained with cut images of melanosomes as input for training data and the mask value of the centre of the cut region as output (Fig. 6C). The mask accuracy for 2000 untrained verification data was almost 97 % after 1000 iterations (Fig. 6D). The 3 % error was considered to be due to manual masking. The DNN trained on the mask image was able to perform melanosome masking with high accuracy (Fig. 6E, F). The DNN learning of masked images and data analysis were calculated using MATLAB (R2021a) with the Image Processing Toolbox and Deep Learning Toolbox. DNN learning of masking data took approximately 25 min on a personal computer (Intel Core i7–9800X, 3.8 GHz and 64 GB RAM, Windows 10).

### 5.8 Image processing of melanosomes

Melanosome images were detected in SE-ADM images of MNT-1 and melanocytes, and the major and minor axis and circularity of each melanosome were calculated and their distribution was obtained (Fig. 7). From seven SE-ADM images (10,000 ×) of MNT-1 on the second day of incubation, 379 melanosome images were automatically detected by the DNN. These images were masked by the DNN and 318 suitable images were manually selected. On day 7 of MNT-1 cell culture, 516 melanosome images 1 were detected in 4 SE-ADM images, and after mask processing, 366 suitable images were selected. For melanocyte images, 631 and 466 melanosome images were detected from 6 and 7 SE-ADM images on the 2nd day and the 5th to 7th days of culture, respectively. For image analysis, 361 and 357 suitable images were manually selected, respectively.

The melanosome images masked by the DNN (Fig. 6F) were subjected to a binarisation analysis to calculate the major and minor axis, and circularity of the particles (Fig. 7A). The above values were calculated using the Matlab command ‘bwconncomp’ by Matlab (R2021a) with Signal processing Toolbox and Image processing Toolbox, which detects and counts connected components in binary images. Origin version 2021a (OriginLab Co., USA) was used to process the distribution images (Fig. 7B−D).

### 5.9 Development of 3D prediction model of melanosomes

A melanosome in an MNT-1 cell is predicted to be toroidal in shape if viewed from a certain direction, while elongated if viewed from a position rotated 90^o^ from that direction. The thickness was set to 170 nm from the particle images of the toroidal structure, and the diameter of the central hole was set to 330 nm from the melanosome toroidal image. The toroidal diameter was taken to be 590 nm from the SE-ADM melanosome images. Based on this structural information, 3D array data of 181 × 181 × 181 voxels was created using Matlab. The 3D model was then rotated using Matlab's 3D display function to create 2D and projection images at each prediction angle.

Melanosomes in melanocytes have an ellipsoidal structure, and from the SE-ADM particle image, the length of the long axis was set to 710 nm and the length of the short axis to 280 nm. Based on this structural information, 3D array data of 100 × 100 × 100 voxels was created using Matlab. The creation of the 2D images were performed in the same way as for MNT-1 using Matlab.

## Funding

This study was supported by 10.13039/501100002241Japan Science and Technology Agency CREST Grant Number JPMJCR19H2, Japan Society for the Promotion of Science KAKENHI Grant Numbers 20K20476, 21K19597, 19H03230 and 19K22442.

## CRediT authorship contribution statement

**T. Okada** and **To. Ogura** designed and performed the research. **To. Ogura** designed and developed the SE-ADM system and culture dish holder. **T. Iwayama** and **S. Murakami** developed the TYR KO MNT-1 and TPC2 MNT-1 cells. All authors contributed to obtaining the experimental data, discussing the experimental results and writing the manuscript.

## Declaration of Competing Interest

The authors declare that they have no known competing financial interests or personal relationships that could have appeared to influence the work reported in this paper.
