# Supplementary material for: Structural analysis of melanosomes in living mammalian cells using scanning electron-assisted dielectric microscopy with deep neural network
Source: Comput Struct Biotechnol J. 2022 Dec 18;21:506–18. doi: 10.1016/j.csbj.2022.12.027 (PMC9807747; doi:10.1016/j.csbj.2022.12.027)
Supplement: Supplementary file 1 — Supplementary material [file mmc1.pdf]

Supplementary data

Structural analysis of melanosomes in living mammalian cells using scanning electron-assisted dielectric microscopy with deep neural network

Tomoko Okada [a], Tomoaki Iwayama [b], Taku Ogura [c], Shinya Murakami [b], Toshihiko Ogura [a,*]

[a] Health and Medical Research Institute, National Institute of Advanced Industrial Science and Technology (AIST), Central 6, Higashi, Tsukuba, Ibaraki 305-8566, Japan

[b] Department of Periodontology, Osaka University Graduate School of Dentistry, 1-8 Yamada-oka, Suita, Osaka 565-0851, Japan

[c] Chemical Business Unit, Nikko Chemicals Co., LTD., Itabashi-ku, Tokyo 174-0046, Japan

*Corresponding author: Toshihiko Ogura
Health and Medical Research Institute, National Institute of Advanced Industrial Science and Technology (AIST),
Higashi 1-1-1, Tsukuba, Ibaraki 305-8566, Japan
Tel.: +81-29-861-3408, Fax: +81-29-861-2677
E-mail: t-ogura@aist.go.jp

This PDF file includes:

Supplementary Figs 1 to 6

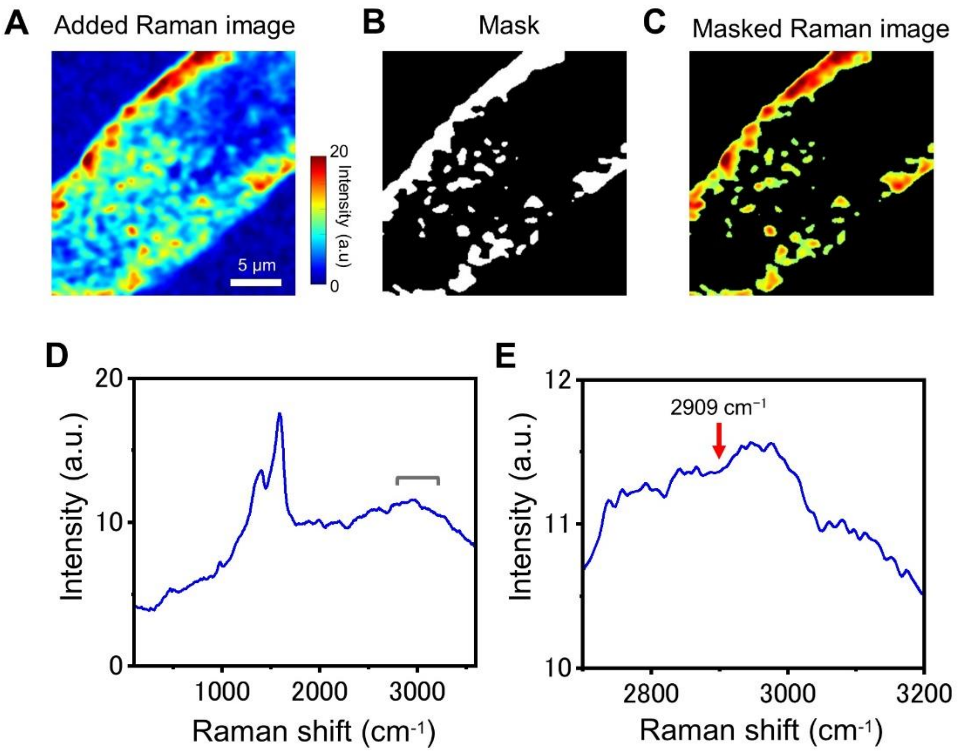

Supplementary Figure 1. Measurement of melanin in MNT-1 cells by laser Raman microscope.

(**A**) An intensity summed image of Fig. 3H and I. It consists of 14,400 pixels of 120 × 120 area. (**B**) A mask image of 2106 pixels selected from (**A**), at which the intensity in arbitrary unit is 10 or more. (**C**) An image of the brightness value of the pixels selected in (**B**). (**D**) An average Raman spectrum of (**C**). Noise is reduced but no lipid peak is seen at 2909 cm^{-1}. Average Raman spectra of (**C**). (**E**) Enlarged Raman spectrum indicated by bracket in (**D**). No lipid peak is detected at 2909 cm^{-1} even after enlargement. Scale bar, 5 µm in (**A**),

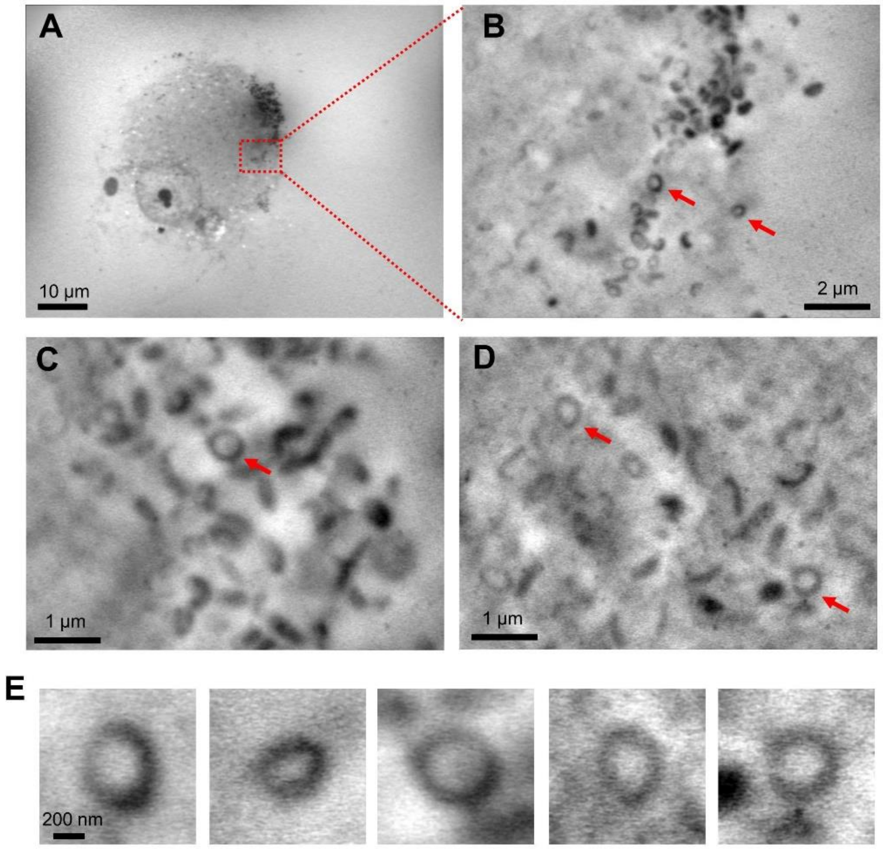

Supplementary Figure 2. Observation of MNT-1 incubated in the medium used for the melanocytes by SE-ADM.

(**A**) A low magnification SE-ADM image (1500×) of living MNT-1 cells incubated in the medium for melanocytes with 8 kV EB. (**B**) A high magnification image (10,000×) of MNT-1 cells in the red rectangle in (**A**). (**C**) and (**D**) Higher magnification images (20,000×) of other areas. (**E**) Enlarged melanosome images indicated by red arrows in (**B**) and (**D**). Scale bars, 10 µm in (**A**), 2 µm in (**B**), 1 µm in (**C**) and (**D**), and 200 nm in (**E**).

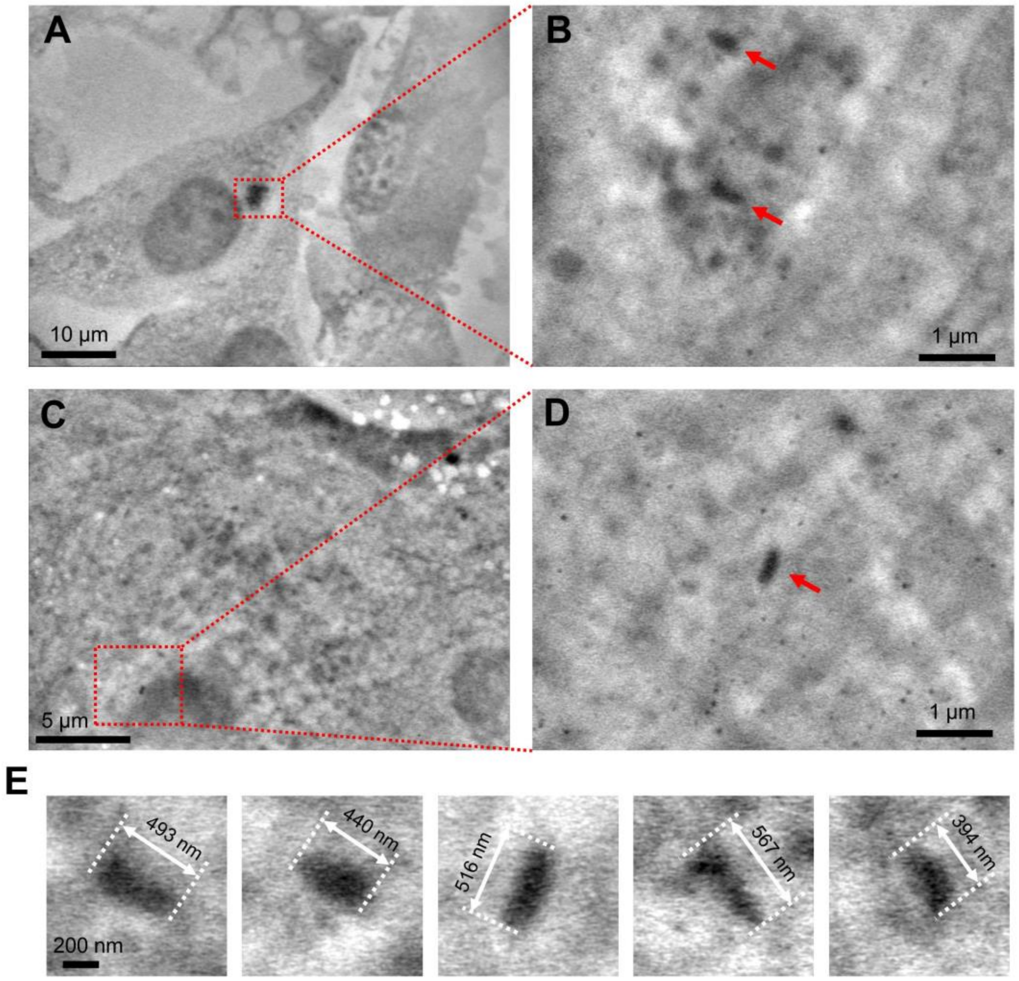

Supplementary Figure 3. Observation of the human adult melanocyte by SE-ADM. (**A**) A low magnification SE-ADM image (2000×) of human adult melanocyte with 8 kV EB. (**B**) An 20,000× magnification image in the red rectangle in (**A**). (**C**) A low magnification SE-ADM image (5000×) of another area. (**D**) A 20,000× magnification image in the red rectangle in (**C**). (**E**) Enlarged melanosome images indicated by three red arrows in (**B**) and (**D**), and another two areas. The average of the major axis of melanosome was 482 ± 67 nm. Scale bars, 10 µm in (**A**), 1 µm in (**B**) and (**D**), 5 µm in (**C**), and 200 nm in (**E**).

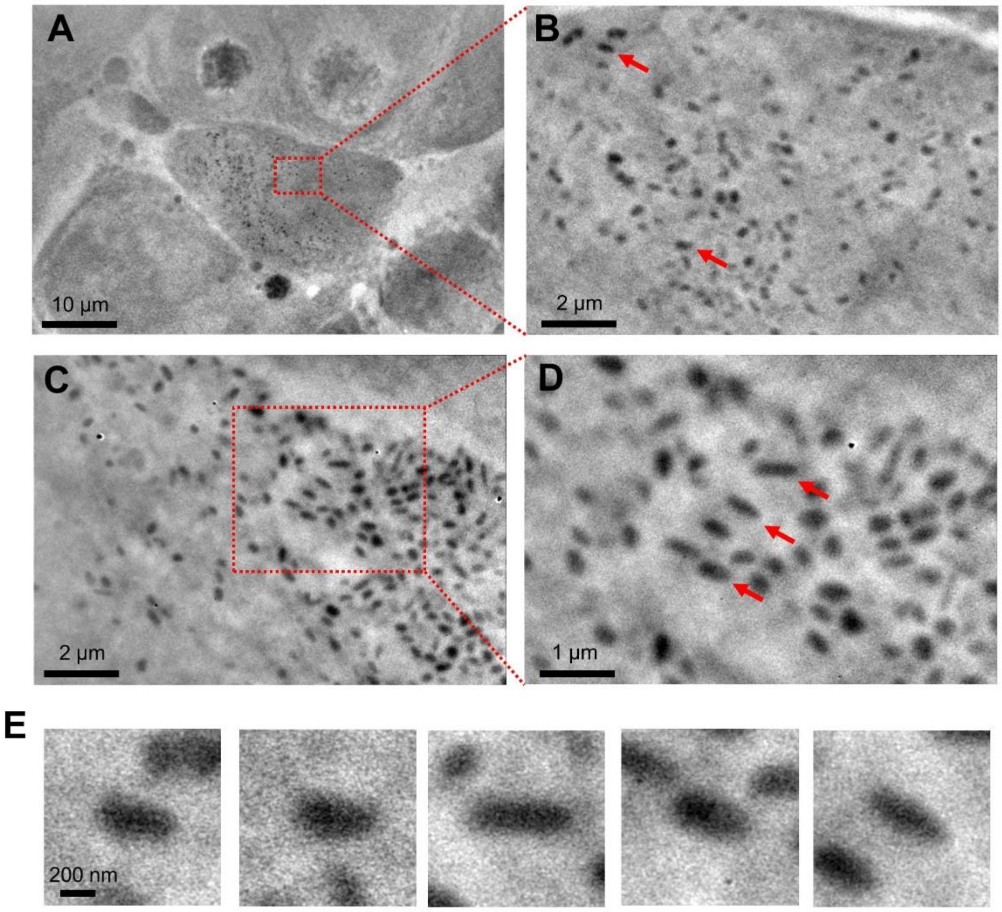

Supplementary Figure 4. Observation of B16 melanoma cells by SE-ADM.

(**A**) A low magnification SE-ADM image (2000×) of B16 melanoma cells with 9 kV EB. (**B**) A high magnification image (10,000×) of B16 melanoma cells in the red rectangle in (**A**). (**C**) A high magnification image (10,000×) of another area. (**D**) A higher magnification image (20,000×) in the red rectangle in (**C**). (**E**) Enlarged melanosome images indicated by the red arrows in (**B**) and (**D**). Scale bars, 10 μm in (**A**), 2 μm in (**B**) and (**C**), and 200 nm in (**E**).

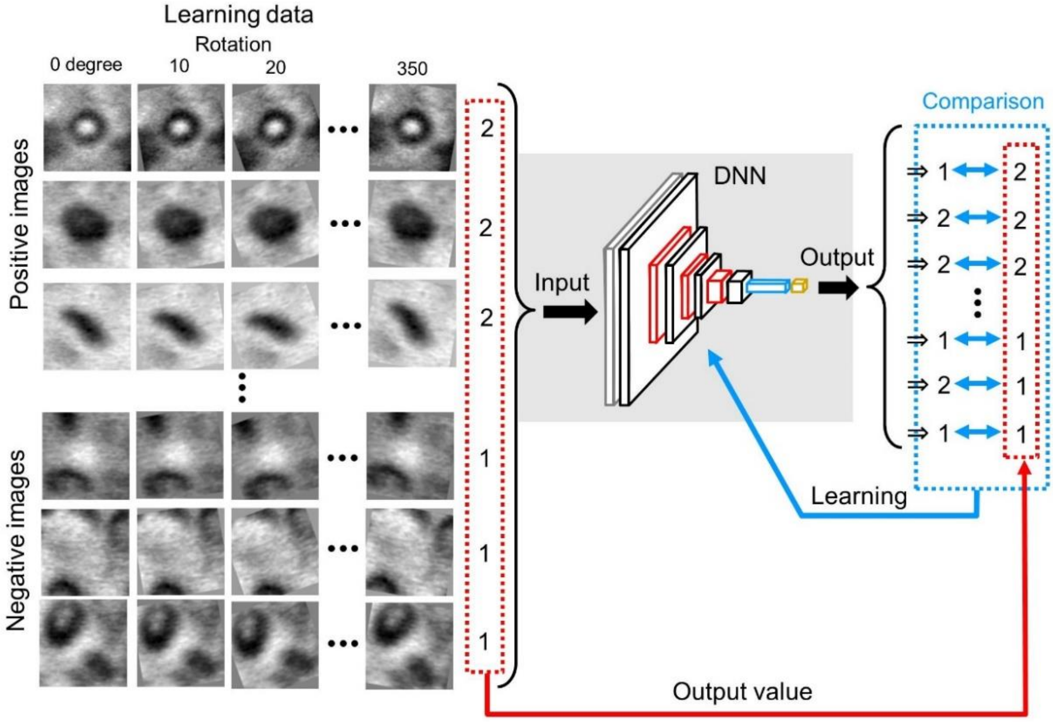

Supplementary Figure 5. Detail schematic diagram of the automatic particle detection system by DNN.

The 239 manually selected melanosome images and 307 background images were rotated in-plane in 10-degree increments to increase the number of training data 36-fold to 19,656. In the input layer of the DNN, these particle images of 100 × 100 pixels were reduced to a size of 50 × 50 pixels and the intensity values were normalized before input.

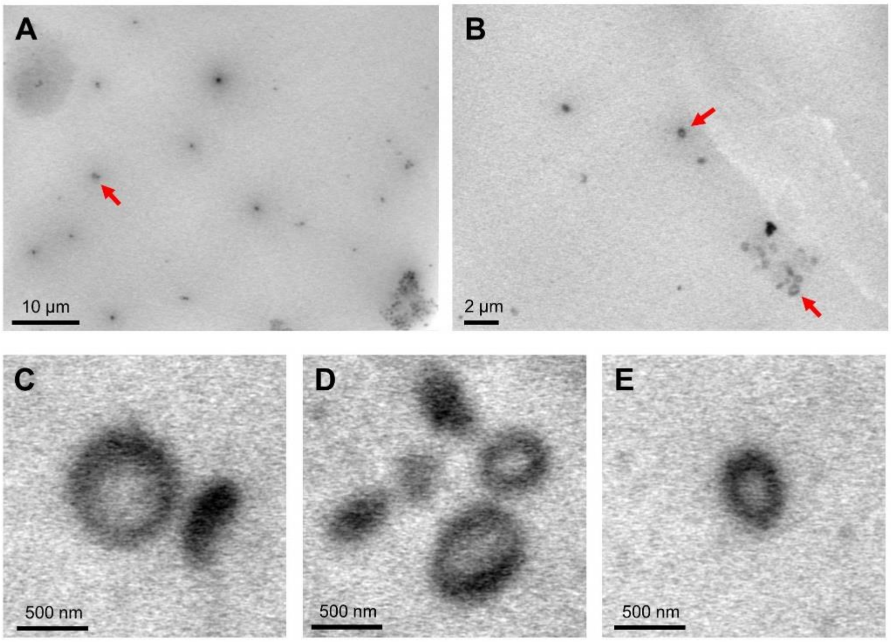

Supplementary Figure 6. Observation of culture supernatant of MNT-1 by SE-ADM. (**A**) SE-ADM image (2000×) of culture supernatant of MNT-1 with 6 kV EB. (**B**) SE-ADM image (5000×) of culture supernatant of MNT-1 with 6 kV EB. (**C**) Enlarged image of melanosomes indicated by a red arrow in (**A**). (**D**) and (**E**) Enlarged image of melanosomes indicated by red arrows in (**B**). Scale bars, 10 μm in (**A**), 2 μm in (**B**) and 500 nm in (**C**)–(**E**).
